# The complete chloroplast genome sequence of *Sloanea leptocarpa* (Elaeocarpaceae) from China

**DOI:** 10.1080/23802359.2022.2078236

**Published:** 2022-05-27

**Authors:** Zhixia Sun, Arief Priyadi, Yanlin Lai, Jianhui Li

**Affiliations:** a College of Architecture Engineering, Sanming University, Sanming, China; b Fujian Provincial Key Laboratory of the Development and Utilization of Bamboo Resources, Sanming University, Sanming, China; c Bali Botanic Garden – Research Center for Plant Conservation, Botanic Gardens, and Forestry, National Research and Innovation Agency (BRIN), Jakarta, Indonesia; d College of Arts and Design, Sanming University, Sanming, China

**Keywords:** *Sloanea leptocarpa*, chloroplast genome, phylogenetic

## Abstract

*Sloanea leptocarpa* Diels, 1931 of the Elaeocarpaceae is an endemic plant from China distributed in subtropical evergreen broad-leaved forests and an excellent ornamental tree. The only available chloroplast genomic resource of the genus at present is that of *S. sinensis* (Hance) Hemsl., 1900 from eastern China. Here, we report the complete chloroplast genome sequence of *S. leptocarpa* which is less common than *S. sinensis*. The complete chloroplast genome of *S. leptocarpa* is 158,077 bp in length, and shows quadripartite organization including a pair of inverted repeat regions (IRs) (24,963 bp) that is divided by a large single-copy (LSC) region (88,519 bp) and a small single-copy (SSC) region (19,632 bp). The circular chloroplast genome of *S. leptocarpa* contains 119 unique genes, composed of 74 protein-coding genes, 37 tRNA genes, and eight rRNA genes. Phylogenetic analysis involving 52 species with complete chloroplast genomes supported that *S. leptocarpa* is closely related to *S. sinensis*. This finding is in agreement with previous studies in which Elaeocarpaceae belongs to Oxalidales instead of Malvales and provides additional evidence for the monophyly of the *Sloanea*, a sister clade of the *Elaeocarpus*.

*Sloanea leptocarpa* Diels., 1931 is among seven endemic *Sloanea* spp. in China with natural habitat in the subtropical zone of broad-leaved evergreen forests at an altitude of 700–1000 m (Tang and Phengklai 2007). This species may reach the height of 20 m and position as a second-layer canopy tree (Wang et al. 2014). *S. leptocarpa* is also an excellent ornamental tree in urban green spaces. The family of Elaeocarpaceae Juss., 1816 consists of 12 genera in which *Elaeocarpus* L. and *Sloanea* L. are the two naturally distributed in China (Tang and Phengklai 2007). South-west China and northern Indo-China are the central diversity of the Old World *Sloanea* (Coode 1983), and the genus *Sloanea* approximately has 50 species with distribution in the Old World and 100 in the New World (Coode 2004). Among *Sloanea*, the only available report of chloroplast genome at present is that of *S. sinensis* from south-eastern China (Weng et al. 2021). Here, we report the complete chloroplast genome sequence of *S. leptocarpa*, which is less common than *S. sinensis*, based on the Illumina sequencing data. This study aimed to characterize the complete chloroplast genome sequence of *S. leptocarpa* as a resource for future genetic studies of this and other related species.

With the permission of South China Botanical Garden, the fresh leaves of *S. leptocarpa* were collected from South China Botanical Garden, Chinese Academy of Sciences, Guangzhou, Guangdong Province, China (Long. 113.3690 E, Lat. 23.1842 N). Voucher specimens of *S. leptocarpa* were deposited at the herbarium of South China Botanical Garden (accession number: SCBG-DSW1717, contact: Chen Feng, fengchen0215@scbg.ac.cn). Total genomic DNA was extracted following the modified CTAB-chloroform protocol (Doyle and Doyle 1990). We sent the total genomic DNA to Novogene-Beijing (Illumina, San Diego, CA) for sequencing (paired-end 150 bp) and building the library (the insertion size of 350 bp) using the Illumina XTen platform. Removing the low-quality reads from the raw data, we assembled the clean reads using the program NOVOPlasty (Dierckxsens et al. 2017). We used a ribulose-1,5-bisphosphate carboxylase/oxygenase (rbcL) gene sequence from *S. sinensis* (GenBank accession no. MW190090) as the seed sequence. To resolve the inverted repeat (IR) in the chloroplast genome of *S. leptocarpa*, we used a reference of the whole chloroplast genome sequences of *S. sinensis* (MW190090). The assembled chloroplast genome was annotated by CpGAVAS (Liu et al. 2012) and GeSeq (Tillich et al. 2017). We deposited the annotated chloroplast genomic sequence in GenBank with an accession number: MZ359674.

The complete chloroplast genome of *S. leptocarpa* is 158,077 bp in length and shows a quadripartite construction with two inverted repeat regions (IRa and IRb) of 24,963 bp that is insulated by a large single-copy (LSC 88,519 bp) region and a small single-copy (SSC 19,632 bp) region. The overall GC content of the whole genome is 37.2%, and the GC content of the LSC, SSC, and IR regions is 35.0%, 31.9%, and 43.2%, respectively. The complete chloroplast genome of *S. leptocarpa* contains 119 unique genes, including 74 protein-coding genes, 37 tRNA genes, and eight rRNA genes. The chloroplast genome length of *S. leptocarpa* is 76 bp larger than that of *S. sinensis* (Weng et al 2021) and with similar GC content.

Phylogenetic analysis was performed to confirm the position of *S. leptocarpa*. We used the complete chloroplast genomes of 52 plant species, including *S. leptocarpa* itself and 51 additional plants from the Malvales, Oxalidales, Malpighiales, Celastrales, Fabales, Cucurbitales, and Rosales, as well as *Tropaeolum pentaphyllum*, *Crateva tapia*, *Capparis spinosa*, the member of the Brassicales, as the outgroup. The complete chloroplast genomes were aligned using Multiple Sequence Alignment based on Fast Fourier Transform – MAFFT (Nakamura et al. 2018) and a maximum-likelihood tree (Figure 1) was constructed using IQ-TREE under best-fit model GTR + G+I based on Bayesian information criterion (BIC) (Nguyen et al. 2015). The phylogenetic analysis result strongly supported that *S. leptocarpa* is closely related to *S. sinensis* in the genus *Sloanea* (Figure 1), and *S. leptocarpa* is a member of the Elaeocarpaceae family which should be placed in Oxalidales instead of Malvales. This finding is consistent with the previous studies on Elaeocarpaceae (Wang et al. 2021; Weng et al. 2021). Our work provides additional evidence for the monophyly of *Sloanea* as the sister clade of *Elaeocarpus* in the Elaeocarpaceae. In conclusion, this *S. leptocarpa* chloroplast genome will provide a genomic resource for future genetic studies and new molecular data to illuminate the Oxalidales evolution.

**Figure 1.**
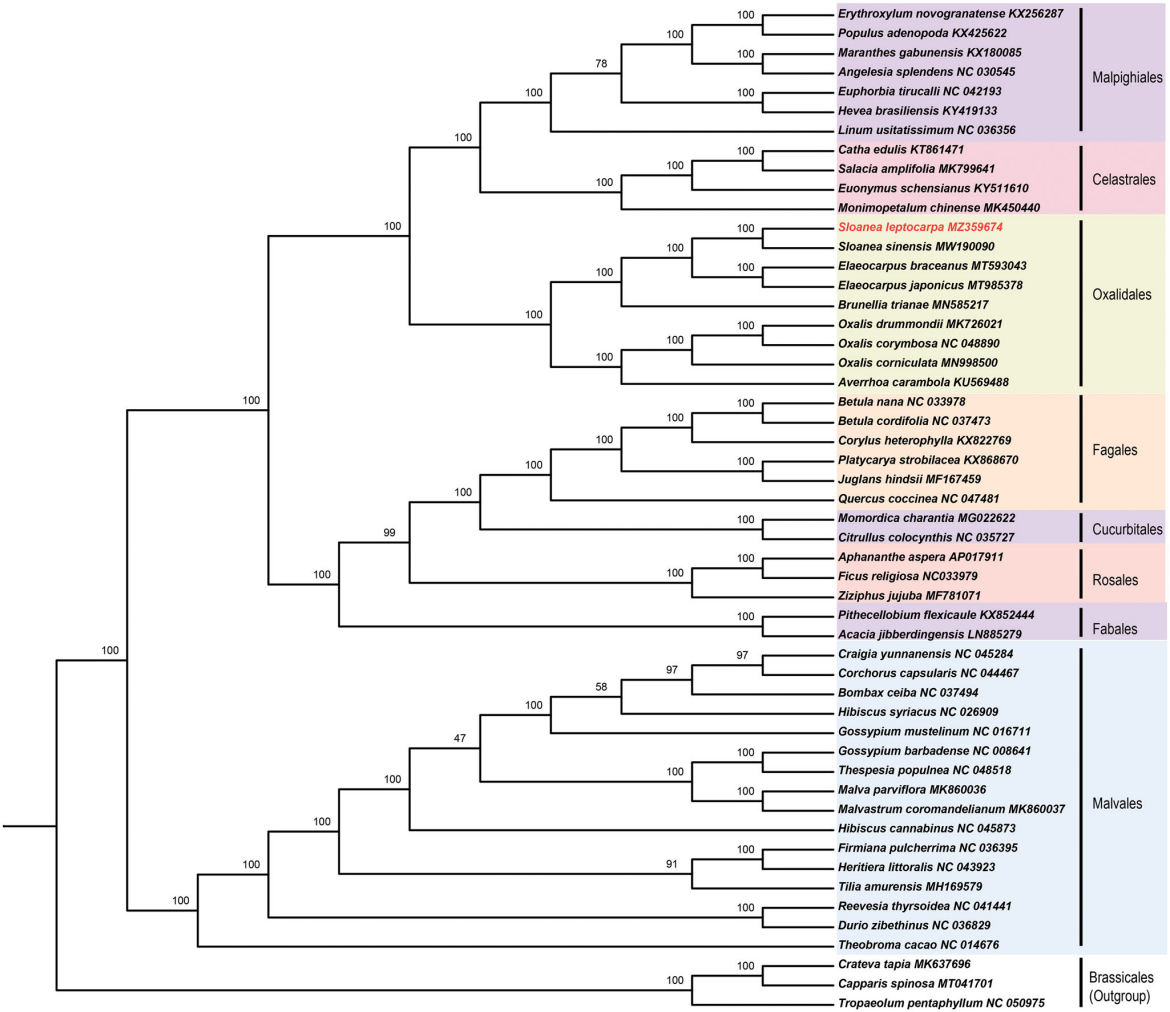
Maximum-likelihood tree based on the 52 chloroplast genomes in Malpighiales, Celastrales, Oxalidales, Fagales, Cucurbitales, Rosales, Fabales, Malvales, and used Brassicales as an outgroup. Bootstrap supports based on 1000 replicates are given at the node.

## Author contributions

ZS conceptualized the research, performed data analyses, and wrote the manuscript. AP contributed to revising the manuscript. YL participated in the data analyses. JL participated conceptualizing the research. All authors made critical contributions to writing the manuscript.

## Data Availability

The genome sequence data that support the findings of this study are openly available in GenBank of NCBI at https://www.ncbi.nlm.nih.gov/, under the accession no. MZ359674. The associated BioProject, SRA, and Bio-Sample numbers are PRJNA763694, SRR15909932, and SAMN21447341, respectively.
